# The effect of intergroup competition outcome on ingroup cooperation: insights from the male warrior hypothesis

**DOI:** 10.3389/fpsyg.2024.1303372

**Published:** 2024-05-24

**Authors:** Montserrat Belinchón, Pablo Polo, Carlos Rodriguez-Sickert, Oriana Figueroa, Nohelia Valenzuela, Paula Pavez, José Antonio Muñoz-Reyes

**Affiliations:** ^1^Laboratorio de Comportamiento Animal y Humano, Centro de Investigación en Complejidad Social, Facultad de Gobierno, Universidad del Desarrollo, Santiago, Chile; ^2^Facultad de Educación, Universidad San Sebastián, Santiago, Chile

**Keywords:** male warrior hypothesis, intergroup conflict, ingroup cooperation, competitive outcome, public good game

## Abstract

**Introduction:**

The Male Warrior Hypothesis (MWH) proposes that sex-specific selective pressures have promoted male cooperation with the ingroup members to outcompete rival groups. However, intergroup conflicts do not occur in isolation and the outcomes of previous competitions may influence group cooperativeness. Since this phenomenon is not well understood, we aimed to shed light on the effect of previous competition outcome on later cooperative behavior under intergroup conflicts. Based on the MWH, we hypothesized that repeated contests between groups could enhance ingroup cooperation, regardless of the outcome of the previous contest because status is at risk, but when competition is not present, participants would move to the symmetric equilibria.

**Methods:**

To test this hypothesis, we recruited 246 individuals organized in groups of 6 and measured cooperation using a threshold public good game over two rounds, manipulating the outcome in the first round to create groups of winners and losers.

**Results:**

Our results show that intergroup conflict scenarios promoted cooperation in both victory and defeat conditions, whereas, in the control scenario only losers increased their cooperation.

**Discussion:**

We argue that winners under the presence of an external threat may enhance in-group cooperation in order to assure their status; whereas, losers may be attempting to regain it.

## 1 Introduction

Humans are adapted to live and cooperate in social groups due to the vast benefits that group living involves, such as division of labor, acquiring and maintaining reproductive resources, or avoiding predators (Tattersall, 2011; McDonald et al., 2012). In parallel, group living came with competition for resources, giving rise to very different patterns of intra and intergroup aggression. Focusing on the latter, competition between social groups has been present since early hominids (Keeley, 2014; Lahr et al., 2016) to modern societies and hunter-gatherer tribes (Chagnon, 1988) as well as in non-human primates (Wrangham and Peterson, 1996). This intergroup competition is thought to have played an important role in human evolution eliciting an intergroup psychology that enables individuals to cooperate with the ingroup members while, at the same time, increasing hostility towards outsiders (Choi and Bowles, 2007; Halevy et al., 2008; Bowles, 2009; Weisel and Böhm, 2015).

Cooperation is a fundamental aspect of human behavior, but its manifestation can vary depending on various social factors (Bowles and Gintis, 2011). Of particular significance is the presence of a competitive environment since, as we already mentioned, there are studies that have found that competition plays a crucial role in shaping cooperative behavior (McDonald et al., 2012). For instance, men exhibit more altruistic and cooperative behaviors toward members of their own group during intergroup conflicts (Stirrat and Perrett, 2012; Muñoz-Reyes et al., 2020), while female cooperation remains unaffected by such scenarios (Van Vugt et al., 2007; Yuki and Yokota, 2009). In an attempt to explain these findings, researchers have proposed the “male warrior hypothesis” (Van Vugt et al., 2007). According to this functional proposal, men have evolved psychological mechanisms that enhance intragroup cooperation during intergroup conflicts because of the substantial benefits derived from aggressive competitions throughout human evolution (Van Vugt and Hardy, 2010; McDonald et al., 2012). This disparity between sexes can be attributed to men’s lower minimum obligatory parental investment, which provides them with higher potential reproductive success and greater advantages resulting from direct competitions (Trivers, 1972; Clutton-Brock and Parker, 1992; Betzig, 2012). In this regard, intergroup contests represent an intrasexual competition scenario to obtain and protect resources that are turned into reproductive resources, such as sexual mates, territory, or social status (Cosmides and Tooby, 1992; Van Vugt and Hardy, 2010; McDonald et al., 2012). However, under some circumstances of social instability, these contests may occur as a series of successive competitions instead of isolated ones because social hierarchies are not defined (Boehm, 2012). In these cases, the outcome of previous competition may affect the motivation and willingness to compete again (Geniole et al., 2017). Then, competition outcome is another potential factor that can impact cooperative behavior in future competitive interactions.

Previous research regarding competition outcomes has been based on the biosocial model of status (Mazur, 1985), which indicates that individuals that win a conflict should be more predisposed to get involved in future competitive interactions to defend social status but, on the contrary, losers should adopt a submissive role in order to prevent future status decline and physical damage (Mazur and Booth, 1998). This theory is consistent with the “winner-loser” effect, which shows that individuals tend to increase their levels of testosterone after winning a contest but decrease them after losing, which in turn shapes status-seeking behaviors increasing or decreasing competitiveness, respectively (Mazur et al., 1992; Archer, 2006; Aguilar et al., 2013; Geniole et al., 2017). Nonetheless, other studies have failed to find the “winner-loser” effect (Gonzalez-Bono et al., 1999; Schultheiss et al., 2005; Mehta and Josephs, 2006), suggesting that changes in testosterone depend on a number of psychological variables that moderate the effect of winning and losing a competition. In this sense, Mehta and Josephs (2006) found that increases in testosterone after competition in losers were related to their willingness to compete again, then, some individuals may attempt to reclaim status after losing a competition. Moreover, losers of close competitions and the unpredictability of social hierarchies increase levels of testosterone and the motivation to compete again (Zilioli et al., 2014; Zilioli and Watson, 2014). Beyond the effect on testosterone levels, winning or losing a contest affects differentially the mood and satisfaction and that may also influence behavior in future competitions through reappraisal of the situation as challenging or threatening (Salvador and Costa, 2009; Leis and Lautenbach, 2020). These theoretical models, as well as most of the empirical data in this field, are based on how the outcome of an individual competition affects testosterone and further aggressive behavior (e.g., Mazur and Lamb, 1980; Elias, 1981; Archer, 2006; Carré et al., 2009; Zilioli and Watson, 2014). However, there is little research on how previous outcomes of intergroup competitions affect directly ingroup cooperation in immediately subsequent conflicts.

Social dilemmas, as the public good game, have been used extensively to study cooperation under laboratory conditions. In turn, a specific type of public good game, the threshold public good game, has been used in some of the previous studies investigating ingroup cooperation within intergroup conflict (e.g., Van Vugt et al., 2007; Stirrat and Perrett, 2012). In this type of public good game, the common pool disappears if the sum of the contributions in the group fails to reach a given threshold. This may represent an appropriate context to study intergroup conflicts towards monopolizable resources (as territories or mates) since failing to reach a certain degree of cooperation and coordination may lead to losing all the potential gains (i.e., losing the contest). In the absence of intergroup competition, Cadsby and Maynes (1999) proposed that Nash equilibrium theory will predict participants’ cooperation in a repeated public good game bound to reaching or not the threshold. Groups of individuals without the possibility of communicating are expected to move towards two symmetric strategies over time in this game. While symmetric pure strategy equilibrium occurs when all participants contribute zero, the symmetric threshold equilibrium occurs when each participant donates just enough to reach collectively the threshold. Hence, when the threshold is not achieved, participants may stop cooperating and may adopt the symmetric pure strategy, whereas when the threshold is achieved, participants are expected to maintain the symmetric threshold equilibrium.

Under intergroup conflict scenarios, the Nash equilibrium may not be appropriate to explain cooperative behavior over time since status is added to the payoff and it acts as a strategic incentive to win. Then, winning (losing) the contest increase (decrease) social status and the utility derived from cooperation. Two previous studies found that, in a repeated public good game with strategic incentives to win, deserved losers tended to increase their donations regardless of the previous individual contribution (Tan and Bolle, 2007; Kiss et al., 2020). This increment in cooperation is somehow contrary to the expectations from the biosocial model of status that postulates that losers should decrease their predisposition to compete again (Mazur and Booth, 1998), and then, to reduce cooperation in the next round, but instead, the increment of cooperation might represent an opportunity for losers to regain status (Daly and Wilson, 1988; Mehta and Josephs, 2006). Results regarding the victory condition were ambiguous: while in one study winners decreased donations (Tan and Bolle, 2007), in the other acted as conditional cooperators (Kiss et al., 2020), which are not expected responses in conventional approaches in which winners increase their competitive motivations (Mazur, 1985; Geniole et al., 2017). Consequently, due to the lack of consistency between empirical data and within theoretical predictions, our goal is to contribute to clarify the effect of competition outcome on male ingroup cooperation under intergroup conflicts.

Considering the postulates of the male warrior hypothesis (Van Vugt et al., 2007), we proposed that a repeated interaction between groups may enhance ingroup cooperation, independently of the previous group’s history of victory or defeat, given the significant benefits for men related to status acquisition during intergroup conflicts. Losers may be attempting to regain their status/limited resources lost in the last interaction by increasing their ingroup cooperation as suggested by Mehta and Josephs (2006) whereas winners, under the presence of this external threat, may enhance cooperation in order to ensure their status/limited resources. This would be especially true under circumstances of unstable or undefined group hierarchies (Zilioli and Watson, 2014) like competition between groups that do not meet each other before. In order to test this general hypothesis, we measured cooperation via the threshold public goods game in an intergroup conflict scenario and a control context to investigate how the competitive outcome (winning or losing the contest and reaching or not the threshold, respectively) influenced cooperation in the next round. We set out the following specific predictions. First, in a intergroup conflict context, we predicted that the high sensitivity of men to this conflict would result in that both groups of winners and losers in the first round would increase their contributions to maintain or reclaim status/limited resources in the second round. On the contrary, in the control context (i.e., without the intergroup conflict scenario), we predicted that participants in groups that did not reach the threshold would decrease their contributions (i.e., move to the pure symmetric strategy), but winners would tend to maintain their contributions (i.e., symmetric threshold equilibrium) according to the Nash equilibria of the game.

## 2 Methods

### 2.1 Participants

A total of 246 young men (age: M = 22.21 years, SD = 3.20 years) were recruited from universities and general population in the Region of Valparaiso (Chile) through public announcements on social networks and in the laboratory website. Participants were organized into groups of 6 members. We intended to form groups in which individuals knew each other in order to gain ecological validity. We excluded four individuals because they did not complete the entire procedure. The main reason for choosing young male adults between 18 and 39 years old is because intrasexual competition is more intense in that period of life (Wilson and Daly, 1985). At the end of the experiment, participants received $15,000 Chilean pesos (CLP) per individual (around $23 USD) for showing up. In addition, they could receive an additional payment of up to $15,000 CPL according to their individual (and group) performance in the game. Most of the participants (90%) received the total amount of $30,000 CLP.

### 2.2 Ethics committee

This experiment, including protocols and data treatment, was approved by the Institutional Bioethics Committee of the Universidad de Playa Ancha (Chile). Participants must have read and signed the informed consent form prior to the experiment. In this document, all the procedure and anonymity protocols were presented. In order to ensure anonymity, we linked all the individuals’ responses to alphanumeric codes.

### 2.3 Procedure

We had two manipulated sets of conditions. First, groups were assigned to an intergroup competition scenario or a control scenario. In the intergroup competition condition, participants were informed that they were playing synchronously against a group of men from another university in the country that was participating in the same project, so the group that reached the threshold faster would win. Only the winners would get the bonus. This group was fictitious to simulate an intergroup competitive scenario with one of three potential universities in the country that differ in their nature as public or private institutions (two public and one private) and in the access score. Participants were not informed which institution were competing against. We included in the potential pool of competitors public and private institutions because regardless of the origin and socioeconomic level of the participants, there would always be a potential competing group with different characteristics. These institutions corresponded to the affiliations of some of the project’s co-investigators, but for logistical reasons, data was only collected at the institution of the principal investigator (a fourth institution). In the control condition, participants were informed that they were playing synchronously among them. They were informed that they could gain a bonus if they reached the threshold as a group. No mention was made of other groups. The second condition was whether the group was assigned to the winner or the loser condition in the first round of the game. This first round of the game was manipulated in terms of the group performance. In the winner condition, groups were informed that they reached a total amount of $20,000 Chilean pesos in their common good and accordingly, they won the bonus regardless of the actual group performance. In the context of intergroup competition, it was added that the group exceeded the threshold (18,000 Chilean pesos) before the rival group. In the loser condition, groups were informed that they reached a total amount of $17,000 Chilean pesos in their common good and accordingly, they lost the bonus regardless of the actual group performance. Individuals only had information about their own contribution and the manipulated contribution of the group. Accordingly, they were unable to know their real performance and therefore doubting the credibility of the group performance. In both conditions, in the second round, the participants were informed about their real performance. That is, groups that exceeded the threshold were informed that they won the bonus. Otherwise, they were informed that did not gain the bonus. Groups were randomly assigned to one of these conditions.

Participants first completed a sociodemographic questionnaire in which they responded to questions about their age, sex, sexual orientation, socioeconomic status, and place of residence. After that, they were informed about the procedure of the threshold public good game in a meeting room. These instructions were provided by a researcher and the protocol of the speech is provided in Supplementary material. The instructions were also provided inside the game (see Supplementary material) before they played a practice game. Then, they played the first round of the game. After the outcome manipulation, they were asked to play a second round in which the outcome was not manipulated. The experiment was conducted in the Laboratorio de Comportamiento Animal y Humano of the University of Playa Ancha (Chile) in six isolated experimental cabins with computers so participants could not communicate with each other. In addition, participants only had information about their individual contributions to the public good. In other words, participants were not able to know their real performance as a group in the first round of the game (the round in which the outcome was manipulated).

### 2.4 Behavioral measure

Cooperation was measured by the threshold public good game. We employed the contribution of the individuals to assess their cooperative behavior (Zelmer, 2003). We followed the protocol established by Van Vugt et al. (2007) and replicated by Stirrat and Perrett (2012) to measure contributions under the presence and absence of intergroup conflict and following winning and losing the first round. Then, participants played two consecutive rounds of the game. The threshold public good game was played on computers using z-Tree software (Fischbacher, 2007). The starting endowment was $5,000 Chilean pesos which they could destinate any amount of that for the group endowment. They were told that if they exceeded a threshold (total invested) of $18,000 Chilean pesos (which would involve a mean cooperation of $3,001, i.e., 60% of their initial endowment), they would receive a bonus of $11,000 Chilean pesos, regardless of the aggregate contributions. In the case of the intergroup conflict scenario, they were told that, in the case that the rival group exceeded the threshold, they needed to reach that objective before the rival group to gain the bonus. On the contrary, if the group did not exceed that threshold, participants would receive just the amount they decided not to contribute. As we have mentioned, the outcome of the first round was manipulated regardless of group performance but participants were not aware of this manipulation. The outcome of the second round depended on whether the group exceeded the threshold in both conditions (intergroup competition and control) since the rival group was fictitious.

### 2.5 Data analysis

To test our predictions, we employed hierarchical linear models (HLM) in order to account for the repeated nature of our data (West et al., 2007). Level-1 variables were those regarding within individual measures and level-2 variables were those regarding between individual measures. To test our first prediction, we selected only data for groups in the intergroup conflict scenario and we fitted a HLM with contributions in the public good game (level-1 variable) as the dependent variable. The round of the game (level-1 variable) and the condition (losing or winning the first game, level-1 variable) were our independent variables. We included the interaction between round and condition. We controlled for age (level-2 variable) and socioeconomic status (level-2 variable). Individual ID was included as a random effect. To test our second prediction, we selected data for groups in the control condition and fitted the same model indicated above. We decided to fit two independent models since comparisons between control and intergroup conflict conditions were published elsewhere (Muñoz-Reyes et al., 2020). For both models, we specified full maximum likelihood estimation and Type III variance. Post-hoc tests (using Bonferroni correction) followed whenever a significant interaction effect was detected. Since hierarchical linear models entail residuals at different levels, we calculated for each significant result the effect size following the following expression:


$${ƒ}^{2}=\frac{R_{2}^{2}-R_{1}^{2}}{1-R_{2}^{2}}$$

where $R_{2}^{2}$ represents the variance explained for a full model and $R_{1}^{2}$ the variance explained for a model in which a given effect was removed (Lorah, 2018). In order to calculate $R^{2}$ of the models, we employed the following expression:


$$R^{2}=1-\frac{\sigma_{F}^{2}+\tau_{F}^{2}}{\sigma_{E}^{2}+\tau_{E}^{2}}$$

where $\sigma_{F}^{2}$ is level-1 error variance of the full model, $\tau_{F}^{2}$ is level-2 error variance of the full model, $\sigma_{E}^{2}$ is level-1 error variance of the empty or null model and $\tau_{E}^{2}$ is level-2 error variance of the empty or null model (Lorah, 2018). The global significance level was set at α = 0.05. Models were performed with IBM SPSS 25 software.

## 3 Results

Table 1 shows mean contributions (and standard deviations) in the threshold public good game in each round according to the competition outcome condition for both intergroup conflict and control conditions.

**Table 1:** Mean contributions and standard deviations (SD) in the threshold public good game according to the round (1st Round vs. 2nd Round), the competition outcome (Defeat vs. Victory) and condition (Intergroup conflict vs. Control).

	Control condition				Intergroup conflict condition			
	Defeat (*N* = 66)		Victory (*N* = 54)		Defeat (*N* = 60)		Victory (*N* = 66)	
	Mean	SD	Mean	SD	Mean	SD	Mean	SD
1st Round	3,185	1,169	3,440	951	3,515	1,236	3,761	948
2nd Round	3,719	1,032	3,403	932	3,760	1,220	3,898	881

**Figure 1:**
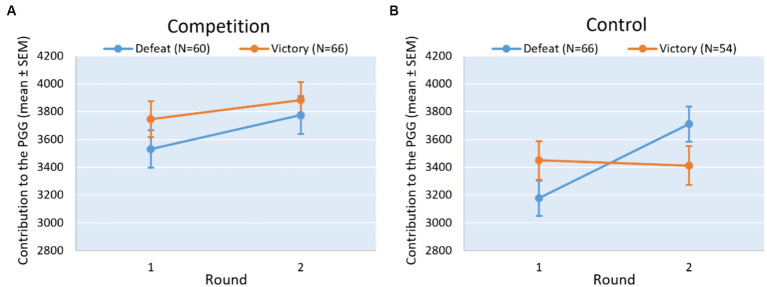
Estimated mean contribution ± standard error of the mean (SEM) in the threshold public good game in the first and the second round for the victory and defeat condition in the intergroup conflict **(A)** and the control context **(B)**. Orange line depicts victory condition and blue line depicts defeat condition.

Regarding our first prediction in which we expected an increase in cooperation in both winners and loser when facing a intergroup conflict condition, the results (Table 2) show that there was no main effect of competition outcome on cooperation, (*F*(1, 126) = 0.84; *p* = 0.360) but there was a main effect of round (*F*(1, 126) = 7.19; *p* = 0.008; *ƒ^2^* = 0.008). Overall, contributions were higher in the second round (estimated mean = 3892.54, SE = 93.72) than in the previous one (estimated mean = 3638.90, SE = 93.72). The interaction effect between round and competition outcome was not significant, (*F*(1, 126) = 0.58, *p* = 0.447), that is, both winners and losers increased their contributions and no difference was found between them in any round (Figure 1A). Finally neither age (*F*(1, 126) = 2.36, *p* = 0.127) nor socioeconomic status (*F*(1, 126) = 3.42, *p* = 0.067) were significant in the model. Since only round was a significant predictor, alternatively we can test its effect with a simpler t-test of repeated measures analysis. In this case, the differences remained significant (*t* = −2.631, *df* = 125, *p* = 0.010, Cohen*’*s *dz* = 0.234).

**Table 2:** Estimated parameters for contributions in the threshold public good game in the intergroup competitive scenario (*N* = 126).

Fixed effect	B	t	*p*-value
Intercept	5517.53	6.797	< 0.001
Condition = 0	−106.20	−0.563	0.574
Round = 0	−136.38	−1.390	0.167
Age	−41.59	−1.536	0.127
SES	−232.32	−1.850	0.067
Condition = 0 * Round = 0	108.50	−0.763	0.447

Covariance parameter	Estimate	SE	ICC
Residual	317862.02	40046.85	0.712
Intercept (ID)	786293.31	120758.72	

Condition [0 = Loser]; Round [0 = First]; SES, Socioeconomic Status.

Regarding our second prediction in which we expected the winners to keep cooperation unchanged, while the losers to decrease it in a control condition, the results (Table 3) show that there was no main effect of competition outcome on cooperation, (*F*(1, 120) = 0.06, *p* = 0.939), but there was a main effect of round, (*F*(1, 120) = 10.91, *p* = 0.001; *ƒ^2^* = 0.031). Overall, cooperation is higher in the second round (mean = 2561.63, SE = 93.48) than in the first one (mean = 3313.23, SE = 93.47). However, interaction effect between round and competition outcome was significant, (*F*(1, 120) = 14.412, *p* < 0.010; *ƒ^2^* = 0.019). Pairwise comparisons showed that the mean contribution was significantly different in losers between the first and second rounds (mean differences = −0.498, *df* = 120, *p* < 0.001) but winners’ contributions did not significantly differ between rounds (mean differences = 0.035, *df* = 120, *p* = 0.740) (Figure 1B). In addition, we found that losers and winners did not differ in their contribution either in the first round (mean differences = −0.254, *df* = 164.23, *p* = 0.149) or in the second round (mean differences = 0.279, *df* = 164.23, *p* = 0.114). Finally, neither age (*F*(1, 120) = 1.67, *p* = 0.199) nor status socioeconomic (*F*(1, 120) = 0.09, *p* = 0.923) were significant in the model.

**Table 3:** Estimated parameters for contributions in the threshold public good game in the control scenario (*N* = 120).

Fixed effect	B	t	*p*-value
Intercept	4186.52	5.665	< 0.001
Condition = 0	298.62	1.590	0.114
Round = 0	37.06	0.332	0.740
Age	−36.74	−1.292	0.199
SES	11.46	0.096	0.923
Condition = 0 * Round = 0	−570.90	−3.796	< 0.001

Covariance parameter	Estimate	SE	ICC
Residual	335841.78	43356.99	0.676
Intercept (ID)	702075.80	114389.08	

Condition [0 = Loser]; Round [0 = First]; SES = Socioeconomic Status.

## 4 Discussion

In this study, we aimed to investigate the role of intergroup competition outcomes on intragroup cooperation. Concretely, we tested for differences in contributions between winners and losers in two consecutive rounds in a threshold public good game in two contexts: competing against a rival group and in the absence of this competition. We found support for our first prediction as, under a competitive scenario, cooperation is heightened in the second round of the game independently of the previous competition outcome. That is, both losers and winners increased their cooperation in the second round when competition with another group was mentioned. However, we only found partial support for our second prediction. As expected, winners did not change their contributions in the second round in the absence of intergroup competition, but against our prediction, losers also contributed significantly more in the second round. These results provide some hints about the strategic use of cooperation under intergroup competitive scenario as predicts the male warrior hypothesis.

The male warrior hypothesis (Van Vugt and Hardy, 2010; McDonald et al., 2012) argues that intergroup conflicts represent an opportunity for men to acquire or defend status. In line with this hypothesis, we proposed in our first prediction that, after a competition, losers may be attempting to regain and acquire their status lost in the last interaction, and winners to defend it. Therefore, we predicted that the competition outcome would not affect ingroup cooperation in a subsequent contest because increasing cooperation has potential benefits for both winners and losers regarding to social status. Our results indicate that male groups increased cooperation in a subsequent competition independently of the outcome of the previous contest supporting our prediction. On the one hand, these results are partially in accordance with studies under similar methodological conditions, that is, using the threshold public good game under an intergroup contest. These studies found that losers increased contributions when their group contributed less than the rival group (deserving losers) (Tan and Bolle, 2007; Kiss et al., 2020). On the other hand, for the victory condition, results differ between studies: while in one study winners acted as conditional cooperators (Kiss et al., 2020) in the other winners decreased contributions with and without monetary incentives (Tan and Bolle, 2007). Both results contrast with the increase in cooperation that we found. A potential reason that may explain the differences between our results and the two mentioned studies is that, in the previous ones, groups were formed by men and women and according to the male warrior hypothesis (Van Vugt and Hardy, 2010; McDonald et al., 2012), group performance could be biased because females are not affected by intergroup conflicts as males are. In addition, in the study of Kiss et al. (2020) the probability of being chosen as the winner was proportional to the performance of the group relative to the other group so the winning group was not always the one that contributed the most. This adds an element of chance that our design did not contemplate and that could be affecting the logic of the competition for a monopolizable resource. And finally, in our study participants played only two rounds whereas in the mentioned studies played 10 and 20 rounds. The behavioral response to win or lose may be different in the first rounds compared to the last ones in a sequence of 10 or 20. In fact, in the study of Tan and Bolle (2007) participants playing with partners and in the intergroup competition condition with incentives seem to increase their contributions regardless of the previous results in the second and third rounds. This resembles our results, unfortunately, this study only reports results from all rounds averaged.

In addition, our results are in contrast to predictions derived from the biosocial model of status (Mazur, 1985), which argues that winners may be involved in further competitions, but losers would tend to withdraw in order to avoid physical aggression and status decline. This theory is in line with the challenge hypothesis (Wingfield et al., 1990; Archer, 2006) which proposes that, under situations of status threat, there are physiological responses associated with testosterone levels that drive dominance-related behaviors producing the “winner-loser” effect: winners increase testosterone levels to reinforce dominant behaviors and losers decrease them (Mazur, 1985). Then, under this model, we might find that winners increase cooperation but losers decrease it because of testosterone levels decline. However, in our study participants played the second round following the first round without any delay, therefore, changes in the cooperation levels cannot be explained by changes in circulating testosterone if we consider that the hormonal effects of winning or losing a competition are delayed 15–20 min (Casto and Edwards, 2016). The increased cooperation among losers can be explained by other psychological factors, such as individual attributions or mood, that also modulate the “winner-loser” effect (Gonzalez-Bono et al., 1999). For example, Kiss et al. (2020) observed that chance losers acted as conditional cooperators but deserved losers increased cooperation, which reflects that attributions related to the outcome may modulate ingroup cooperation. Moreover, circumstances of social instability may lead to a reverse “winner-loser” effect (Geniole et al., 2017), which may promote status-seeking behavior in losers who would be involved in future competitions to reclaim status (Daly and Wilson, 1988; Mehta and Josephs, 2006). Then, when social hierarchies are not defined, as when groups that have not interacted before, like in our study design, we can expect a different tendency for losers (Zilioli and Watson, 2014).

Regarding our second prediction, we proposed that the Nash equilibrium would explain participant’s behavior in consecutive games in a control condition without intergroup competition (Cadsby and Maynes, 1999). In this sense, participants that do not overcome the threshold will move to the symmetric pure equilibrium, but winners will tend toward the symmetric threshold equilibrium. Our results show that participants in the victory condition maintained contributions to overcome the threshold using the previous successful strategy, suggesting that they moved to the symmetric threshold equilibrium. However, participants in the defeat condition tended to behave oppositely as expected by the symmetric pure strategy: they increased contributions. This finding could be explained partly considering the utility and the payoffs from the game because when participants overcome the threshold, they receive a monetary incentive that promotes cooperation. Furthermore, social identity is known to decrease “free-riding” in social dilemmas as participants try to maximize ingroup outcomes (Simpson, 2006). Then, a collective goal—as the threshold in this case—may enhance intrinsic motivation to cooperate and succeed in the group objective even without monetary incentives.

The effect of intergroup conflict on cooperation has taken attention recently because there is robust evidence that cooperation is exacerbated in groups of men when competing against same-sex rival groups (McDonald et al., 2012; Stirrat and Perrett, 2012; Muñoz-Reyes et al., 2020). Nonetheless, intergroup conflicts are more complex than have been represented in experimental settings. Firstly, in some cases, competitions do not occur isolated as most of the time humans become revenge-seekers (Boehm, 2012). Moreover, competition outcome may influence and regulate social hierarchies affecting status and, therefore, further status-seeking behaviors and social interactions (Sidanius and Pratto, 2001; Geniole et al., 2017). In this sense, considering previous competition outcomes will help us to understand more precisely the role of intergroup conflict scenarios on cooperation. However, our results can only be extended to the mentioned competitive settings. For instance, face-to-face contests could lead to different predictions as physical threat could be present and participants have the opportunity to evaluate their rivals’ features defining social hierarchies, which is known to play a key role in modulating competitive behavior (Flinn et al., 2012). Then, under this scenario, there are some possibilities that losers would decrease their competitive behavior (Mazur, 1985). In addition, in our study, the contest was compounded by two games. However, there is evidence indicating that cooperation declines when the game is played in repeated interactions (e.g., Burton-Chellew and West, 2021). It would be relevant to investigate whether the effect found in this study is sustained across rounds and, therefore, if the intergroup conflict scenario is a key element in sustaining cooperation. It might be also possible that further rounds would define group hierarchies so that losers would admit their defeat and winners would not perceive the contest as a challenge, therefore, adopting different competitive behaviors. In addition, previous results showed that cooperation in the public goods game also depends on males’ sexually selected traits (Muñoz-Reyes et al., 2020). Then, it would be valuable also to test in future studies if individual differences may modulate the influence of competition outcome on cooperation. In this study the interaction with the other group was simulated and accordingly, participants lacked a relative measure of their performance compared to the rival’s group performance. This information may be relevant since individuals may change their competitive strategy from more aggressive to more avoidance-oriented according to the formidability (the ability to inflict costs) of the rival group (McDonald et al., 2012). In this sense, if the performance of the rival group is a proxy of their formidability, this information may affect the degree of in-group cooperation from the participants. Future studies in which this information is provided either because there is a real interaction between the groups, or because although it is simulated also simulates different degrees of performance of the other group, would be interesting to understand this problem more deeply. Another limitation is that our study only considered groups of men thus limiting the interpretation of our results to the specific context of intergroup competition between them. It would be relevant in future studies to include groups of women and mixed-sex groups and consider different combinations in the composition of competing groups to gain a deeper understanding of the dynamics of cooperation in intergroup competition contexts. This is because it has been found that both the sex composition of the group and that of the rival group are relevant to the emergence of intergroup discrimination in cooperation and outgroup bias in both men and women (Navarrete et al., 2010; Balliet et al., 2014). Furthermore, recent evidence suggests that group composition is more relevant than sex in revealing differences in cooperation when comparing an intergroup conflict and a control scenario (Muñoz-Reyes et al., 2023). Finally, individuals were not randomly assigned to groups since we aimed to form groups of individuals who knew each other beforehand to gain ecological validity. However, this may introduce potential confounding biases that limit the scope of our results in explaining the underlying mechanisms associated with the male warrior hypothesis.

In conclusion, this is the first attempt to understand how competition outcome affects male ingroup cooperation to outcompete a rival group within the male warrior hypothesis framework. We found that both winners and losers increased cooperation in the second round of a threshold public good game compared with the first round when competition with another group is present. These results suggest that under an intergroup conflict scenario, both winners and losers have incentives to increase cooperation supporting the main postulate of the male warrior hypothesis. We propose that the incentives to cooperate might be driven by a mechanism related to resource monopolization and status-seeking behavior: winners would defend status and losers would try to regain it. This study supports that intergroup conflicts influence male behavior similarly in winners and losers and suggest that an intergroup conflict scenario may enhance ingroup cooperation.

## Data availability statement

The datasets presented in this study can be found in online repositories. The names of the repository/repositories and accession number(s) can be found below: https://osf.io/4p69d/?view_only=811e7d5d2e4147dd92ac1d95efb7ed8c.

## Ethics statement

The studies involving humans were approved by Institutional Bioethics Committee of the Universidad de Playa Ancha (Chile). The studies were conducted in accordance with the local legislation and institutional requirements. The participants provided their written informed consent to participate in this study.

## Author contributions

MB: Formal analysis, Investigation, Writing – original draft, Writing – review & editing. PPo: Conceptualization, Data curation, Formal analysis, Investigation, Writing – original draft, Writing – review & editing. CR-S: Writing – review & editing. OF: Investigation, Writing – review & editing. NV: Investigation, Writing – review & editing. PPa: Investigation, Writing – review & editing. JM-R: Conceptualization, Funding acquisition, Investigation, Methodology, Project administration, Writing – review & editing.
