# Supplementary material for: The effect of intergroup competition outcome on ingroup cooperation: insights from the male warrior hypothesis
Source: Front Psychol. 2024 May 24;15:1303372. doi: 10.3389/fpsyg.2024.1303372 (PMC11157109; doi:10.3389/fpsyg.2024.1303372)
Supplement: Supplementary file 1 [file Table_1.DOCX]

ANNEXES

ANNEX 1. GAME INSTRUCTIONS

The text in square brackets corresponds to the intergroup competition scenario.

Regarding the instructions, they were provided twice: one before they entered the booths to play the game, and the second was provided inside the game on the screen of their computers. After the participants responded to the questionnaires in the booths, they were gathered in another room around a meeting table. Then a researcher informed the participants about the instructions of the game. The speech was “Now you are going to play a game as a group [and against another group that is conducting the same procedure in one of the other three universities that are participating in this research. These universities are Universidad de Santiago de Chile, Universidad Metropolitana de Ciencias de la Educación and Universidad del Desarrollo]. You are going to play three rounds of the game, the first round is a practice round and the other two are real rounds. In each round, you have available 5000 Chilean pesos, and you need to decide how much of this money, if any, you want to contribute to a common good. You can donate any whole amount between 0 and 5000. If you as a group exceed a common amount of 18000 [and you reach this objective before the rival group], each of you will get a bonus of 11000 and you keep the money you decided not to contribute. In the case that you as a group do not reach a common amount that exceeds 18000 [or that the other group achieves that goal before you], you will only receive the amount you decided not to contribute [and the rival group will receive the bonus]. You start each round with 5000 Chilean pesos, that is, the benefits you get in the first round do not accumulate for the second. Any doubts?” Here, participants were allowed to ask questions. After that, participants were informed that from that moment they were not able to communicate among them and that when entering the booth, they were going to see a waiting screen. In the intergroup conflict condition, they were informed that the other group was still ready or that we needed to wait a little for the other group. For both conditions, we informed them that they would see the screen changing when we launched the game. And that on the first screen, they need to provide their ID numbers. Finally, we informed them that sometimes they would have to wait between screens since the game progressed synchronously for everyone. Once, the game was launched and they introduced their ID, the game provided again similar instructions about the game. These instructions are provided below after being translated from Spanish

**Control condition**

Instruction screen 1:

You will participate in two economic games, in each of them you will start your participation with $5000.

The games are independent of each other, so the money you earn in one game WILL NOT ACCUMULATE TO THE NEXT GAME.

Press CONTINUE to advance.

Instruction screen 2:

At the start of the experiment you have $5,000 to play with.

You and the other five participants must exceed a common amount of more than $18,000, to do so you must decide whether you want to donate or not, and thus exceed the amount.

If your donations EXCEED $18,000, you will be given an extra $11,000 bonus to what you had left after the donation.

If the common amount IS NOT EXCEEDED, you will not receive the bonus and you will keep the remainder after your donation.

Outcome screen 1 (losing condition)

Your donation to the group: XXXX (real amount)

The sum of all the group's donations was: 17000 (fixed and manipulated amount)

Your remainder after the donation is: XXXX (real amount)

Outcome screen 2 (losing condition)

IN THIS GAME YOU DID NOT REACH MORE THAN $18,000!

SO YOU DO NOT RECEIVE A BONUS

Outcome screen 1 (winning condition)

Your donation to the group: XXXX (real amount)

The sum of all the group's donations was: 20000 (fixed and manipulated amount)

Your remainder after the donation is: XXXX (real amount)

Outcome screen 2 (winning condition)

CONGRATULATIONS!

YOUR BONUS IS $11000

Your total profit is: XXXXX (remainder + bonus)

**Experimental condition**

Instruction screen 1:

From this moment on, you and your group are part of the network of Universities participating in this Fondecyt project (three public and one private). All the games in which you will participate are being run simultaneously with groups from those Universities to compete with you. The general instructions for the games will be explained below.

You will participate in two economic games, in each of them you will start your participation with $5000.

The games are independent of each other, so the money you get in one game WILL NOT ACCUMULATE FOR THE NEXT GAME.

Press CONTINUE to advance.

Instruction screen 2:

At the start of the experiment you have $5,000 to play with.

You and the other five participants must exceed a common amount of more than $18,000, to do so you must decide whether you want to donate or not, and thus exceed the amount.

If your donations EXCEED $18,000, you will be given an extra $11,000 bonus to what you had left after the donation.

If the common amount IS NOT EXCEEDED, you will not receive the bonus and you will keep the remainder after your donation.

Please note that you will be competing with a group from another university, so the group that exceeds the amount first will win. If both groups reach the amount at the same time, the team that has donated more will receive the bonus.

Outcome screen 1 (losing condition)

Your donation to the group: XXXX (real amount)

The sum of all the group's donations was: 17000 (fixed and manipulated amount)

Your remainder after the donation is: XXXX (real amount)

Outcome screen 2 (losing condition)

IN THIS GAME YOU DID NOT MANAGE TO EXCEED $18000 BEFORE THE RIVAL GROUP!

SO YOU DO NOT RECEIVE A BONUS

Outcome screen 1 (winning condition)

Your donation to the group: XXXX (real amount)

The sum of all the group's donations was: 20000 (fixed and manipulated amount)

Your remainder after the donation is: XXXX (real amount)

Outcome screen 2 (winning condition)

¡ CONGRATULATIONS ON THIS GAME YOU SURPASSED $18000 BEFORE THE RIVAL TEAM!

YOUR BONUS IS $11000

Your total profit is: XXXXX (remainder + bonus)
